# Associations of hearing and visual loss with cognitive decline and dementia risk: a 25-year follow-up of the Maastricht Aging Study

**DOI:** 10.1093/ageing/afae271

**Published:** 2024-12-18

**Authors:** Lion M Soons, Kay Deckers, Huibert Tange, Martin P J van Boxtel, Sebastian Köhler

**Affiliations:** Mental Health and Neuroscience Research Institute (MHeNs), Alzheimer Centrum Limburg, Department of Psychiatry and Neuropsychology, Maastricht University, Maastricht, the Netherlands; Mental Health and Neuroscience Research Institute (MHeNs), Alzheimer Centrum Limburg, Department of Psychiatry and Neuropsychology, Maastricht University, Maastricht, the Netherlands; Care and Public Health Research Institute (CAPHRI), Department of Family Medicine, Maastricht University, Maastricht, the Netherlands; Mental Health and Neuroscience Research Institute (MHeNs), Alzheimer Centrum Limburg, Department of Psychiatry and Neuropsychology, Maastricht University, Maastricht, the Netherlands; Mental Health and Neuroscience Research Institute (MHeNs), Alzheimer Centrum Limburg, Department of Psychiatry and Neuropsychology, Maastricht University, Maastricht, the Netherlands

**Keywords:** hearing loss, visual loss, cognitive decline, dementia, risk reduction, older people

## Abstract

**Background:**

Hearing loss (HL) and visual loss (VL) are recently identified as promising dementia risk factors, but long-term studies with adequate control of other modifiable dementia risk factors are lacking. This 25-year follow-up study investigated the association between objectively measured HL and VL with cognitive decline and incident dementia.

**Methods:**

1823 participants (age 24–82 years) of the Maastricht Aging Study were assessed at baseline, 6, 12 and 25 years. Baseline HL was defined as pure-tone hearing loss ≥20 dB at frequencies of 1, 2 and 4 kHz and VL as binocular, corrected visual acuity <0.5. Associations with cognitive decline (verbal memory, information processing speed, executive function) and incident dementia were tested using linear mixed models and Cox proportional hazard models, respectively. Analyses were adjusted for demographics and 11 modifiable dementia risk factors (LIfestyle for BRAin health index).

**Results:**

Participants with HL (*n* = 520, 28.7%) showed faster decline in all cognitive domains than participants without HL. No consistent association was found for VL (*n* = 58, 3.2%), but below-average visual acuity (<1) showed significant associations with information processing speed and executive function. No significant associations with dementia risk were found. Findings were independent of demographics and modifiable dementia risk factors.

**Conclusions:**

HL predicts faster cognitive decline but not dementia risk in adults aged 24–82 years. VL shows no consistent associations, though below-average visual acuity is linked to faster cognitive decline. This study supports HL as an independent risk factor for cognitive decline. Future studies should further evaluate the roles of HL and VL in dementia risk reduction.

## Key Points

- We examined the association between objectively measured hearing and visual loss with cognitive decline and incident dementia over 25 years.
- Hearing loss (pure-tone hearing loss ≥20 dB) but not visual loss (binocular visual acuity <0.5) was associated with faster cognitive decline across multiple domains.
- No association with incident dementia was found.
- Results were independent of other modifiable dementia risk factors.

## Introduction

The number of people living with dementia is expected to rise due to increasing life expectancy worldwide [1]. Alongside finding disease-modifying treatments, dementia risk reduction through modification of risk and protective factors has therefore become a global health priority [2]. The 2024 Lancet Commission report on Dementia Prevention, Intervention and Care indicated that ~45% of dementia cases are potentially preventable by addressing 14 risk factors [3].

Hearing loss (HL) and visual loss (VL) have gained increasing interest as potential modifiable risk factors for dementia. Globally, HL and VL affect 1.5 billion and 2.2 billion people, and the prevalence increases with age [4, 5]. Moreover, HL and VL resulting from preventable causes like loud noise exposure and eye infections are common [4, 5]. Several systematic reviews have reported a positive association between HL [6–9] and VL [10–13] with risk of cognitive impairment and dementia. A recent review and Delphi expert consensus study found good consistency between the literature and expert opinion for HL as a dementia risk factor, whereas for VL, fairly consistent results from the literature were not supported by expert opinions [14]. HL has been recognised as a modifiable dementia risk factor in midlife by the Lancet Commission since 2017, and in its 2024 report, also VL is acknowledged as a significant dementia risk factor [3], underscoring the growing recognition of both factors in dementia risk reduction.

Yet, many studies have relatively short follow-up periods, increasing the likelihood of reverse causation, or account for only few other modifiable dementia risk factors, leading to residual confounding by factors such as depression [15], cardiovascular risk factors [16, 17] and physical activity [18, 19].

Therefore, this prospective cohort study aims to investigate the association between objectively measured HL and VL with cognitive decline and dementia incidence during 25 years of follow-up and whether these associations are independent of other modifiable dementia risk factors.

## Methods

### Study sample and design

The Maastricht Aging Study (MAAS) is a prospective cohort study on cognitive ageing [20]. In total, 10 396 individuals were invited from the Research Network Family Medicine Maastricht (RNFM), a patient register of collaborating general practitioners representing the Dutch general population [21]. Exclusion criteria included evidence of major neurological conditions or psychiatric disorders that interfere with normal cognitive function. Subsequently, a subsample of 1823 individuals was selected using an optimal stratified sampling design with equally balanced strata for age, sex and level of occupational achievement (low/high). At baseline, participants’ ages ranged from 24 to 82 years, with a median age of 50. Half of the participants were between 36 and 65 years (interquartile range). Participants were evenly distributed between genders (50% female, 50% male). Participants were comprehensively assessed for medical status, lifestyle, and anthropomorphic and neurocognitive measures at baseline, 6, 12 and 25 years [20]. The local ethics committee of Maastricht University Medical Center+ approved the study (MEC05-107), and all participants provided informed consent.

### Hearing loss

Hearing acuity was assessed using pure-tone audiometry thresholds measured with a screening audiometer (Interacoustics AS7, Denmark), testing each ear at frequencies of 0.5, 1, 2 and 4 kHz. Detection thresholds in decibels (dB) were averaged for the better, unaided ear at 1, 2 and 4 kHz, following recommendations for assessing hearing handicap [22]. Higher detection thresholds indicated poorer hearing acuity. Baseline HL was defined as hearing acuity ≥20 dB, according to World Health Organization (WHO) guidelines [4].

### Visual loss

Binocular distance visual acuity was measured with a standard Landolt-C optotype chart at 5 m, including optical correction worn during the examination. Participants identified the orientation of openings in black circles on a white sheet (up, down, left, right). If correctly identified, the circle size decreased. Visual acuity was measured by the size of the smallest circle whose orientation was correctly identified, expressed as the ratio of 5 m to the distance at which a reference group with normal vision can identify the orientation. Higher scores indicated better visual acuity, with a score of 1 considered average for young individuals with normal vision. VL was defined as visual acuity worse than 5/10 (0.5) in both eyes, following WHO guidelines [5].

### Cognitive decline and dementia

At baseline, 6-, 12- and 25-year follow-up, psychologists and trained test assistants administered neuropsychological tests. Verbal memory was assessed using the Visual Verbal Learning Test delayed recall, where participants recalled 15 unrelated monosyllabic words presented on a computer screen during immediate recall (five trial) and delayed recall (20-min delay) [23]. Executive function was measured using the shifting score of the Concept Shifting Test, calculated by subtracting the average completion time of parts A (crossing out digits in ascending order) and B (letters in alphabetic order) from part C (alternating digits and letters) [24]. Information processing speed was assessed with the Letter Digit Substitution Test, where participants matched digits to letters within 90 s, using a reference key [25]. Dementia cases were identified using International Classification of Primary Care codes obtained from the RNFM or diagnosed by a neuropsychiatrist at Maastricht University Medical Center+ based on neuropsychological and clinical data.

### Covariates

Covariates included age, sex, educational level (low, middle, high) and a well-validated composite score (LIfestyle for BRAin health; LIBRA) of nine modifiable dementia risk factors and three protective factors. Detailed information on LIBRA can be found in Appendix 1 in the supplementary material.

### Statistical analyses

Baseline differences between participants with and without HL and VL were assessed by independent samples *t*-tests and *χ*^2^ tests. The Visual Verbal Learning Test score needed square root transformation. Linear mixed models were used to test associations between HL, VL and change in cognitive function over time (verbal memory, information processing speed and executive function). As indicated by likelihood ratio testing, models including a random intercept and slope, with an unstructured covariance matrix, were used for all analyses except for HL and executive function, where an independent covariance matrix was used. Interaction terms (HL × time; VL × time) were included with time as a dummy variable for the follow-ups (1 = baseline to 6 years; 2 = baseline to 12 years; 3 = baseline to 25 years) and tested using *χ*^2^ tests with 3 degrees of freedom. Analyses were adjusted for age, age^2^, sex and educational level, with the fully adjusted model including LIBRA scores and their interaction with time. Results of the fully adjusted model are presented in Tables 2 and 3, and full results in Supplementary Tables S2 and S3. For sensitivity analyses, three-way interactions were tested in the fully adjusted model using *χ*^2^ tests with 3 degrees of freedom to examine potential effect modification by age group (midlife: <65 years at baseline; late life: ≥65 years at baseline), followed by stratified analyses.

The association of HL and VL with incident dementia was assessed with Cox proportional hazard models, computing hazard ratios (HRs) and 95% confidence intervals (CIs). Models included age (on the time axis) and sex (model 1), with subsequent additions of educational level (model 2) and LIBRA scores (fully adjusted model 3, for which results are shown). Sensitivity analyses included age group and its interaction with HL/VL in the fully adjusted model. Dementia was treated as the failure event, with survival time defined as the period from birth until dementia diagnosis, last follow-up or death, whichever came first. The Schoenfeld Residuals Test assessed the proportional hazard assumptions. To address selection bias, a sampling weight was incorporated into all models, allowing estimates to be weighted back to the RNFM population. This gave greater weight to participants less likely to be sampled. Stata 17 (StataCorp, TX) was used with an alpha level of 0.05 in two-sided tests.

## Results

### Sample characteristics

Seven out of 1823 participants did not undergo visual assessment, and nine had missing hearing acuity data at baseline (Table 1). Participants with HL (*n* = 520, 28.7%) were on average older, more often male, less educated, had poorer visual acuity, higher LIBRA scores (indicating unhealthier lifestyles) and lower performance on all neuropsychological tests than those without HL (*n* = 1294, 71.3%). Participants with VL (*n* = 58, 3.2%) were on average older, less educated, had poorer hearing acuity and lower performance on all neuropsychological tests than those without VL (*n* = 1758, 96.8%).

**Table 1:** Baseline characteristics by hearing loss and visual loss status

Baseline characteristics	Total sample	Hearing loss *n* = 1814^a^			Visual loss *n* = 1816^b^		
	*n* = 1823	No, *n* = 1294	Yes, *n* = 520	*P* value	No, *n* = 1758	Yes, *n* = 58	*P* value
Age, mean (SD)	51.1 (16.4)	45.1 (14.4)	66.0 (10.4)	<.001	50.5 (16.2)	69.1 (12.9)	<.001
Female, *n* (%)	910 (49.9)	696 (53.8)	212 (40.8)	<.001	870 (49.5)	34 (58.6)	.171
Educational level, *n* (%)				<.001			.002
Low	665 (36.5)	394 (30.5)	267 (51.5)		628 (35.8)	34 (58.6)	
Middle	746 (41.0)	566 (43.7)	178 (34.4)		726 (41.3)	17 (29.3)	
High	410 (22.5)	334 (25.8)	73 (14.1)		402 (22.9)	7 (12.1)	
Hearing acuity, mean (SD)	14.3 (14.1)	6.8 (5.5)	33.1 (11.3)	<.001	13.9 (13.8)	27.9 (17.3)	<.001
Visual acuity, mean (SD)	1.2 (0.4)	1.3 (0.4)	1.0 (0.4)	<.001	1.2 (0.4)	0.3 (0.1)	<.001
LIBRA score, mean (SD)	0.8 (2.3)	0.7 (2.2)	1.1 (2.5)	.003	0.8 (2.3)	0.6 (2.2)	.546
Neuropsychological tests, mean (SD)							
Verbal Learning Test (raw)	9.7 (3.0)	10.3 (2.8)	8.2 (3.1)	<.001	9.7 (3.0)	7.8 (2.9)	<.001
Verbal Learning Test (transformed)	102.5 (56.6)	113.2 (55.4)	76.1 (50.4)	<.001	103.6 (56.6)	68.9 (48.4)	<.001
Letter Digit Substitution Test (raw)	48.3 (11.8)	51.4 (10.9)	40.5 (10.0)	<.001	48.7 (11.6)	36.9 (8.7)	<.001
Concept Shifting Test (raw)^c^	12.2 (11.6)	10.5 (9.8)	16.3 (13.6)	<.001	11.9 (11.3)	20.3 (16.1)	<.001

Percentages may not sum up to 100% because of rounding errors. *Abbreviations*: SD, standard deviation; LIBRA, LIfestyle for BRAin health index (theoretical range −4.2 to +12.7).

^a^Participants with available baseline hearing acuity data.

^b^Participants with available baseline visual acuity data.

^c^For the Concept Shifting Test, a higher score indicates lower performance.

A total of 313 participants were excluded from the survival analyses because they only had baseline data and their dementia status at follow-up was unknown. After a median follow-up period of 18.6 years (*n* = 1510, mean age: 50.2), 146 participants (9.7%) developed dementia. Back weighting the RNFM source population yielded an incidence rate of 61 cases per 10 000 person-years (95% CI 51.35 to 73.85).

### Hearing loss

#### Hearing loss and cognitive decline

**Figure 1:**
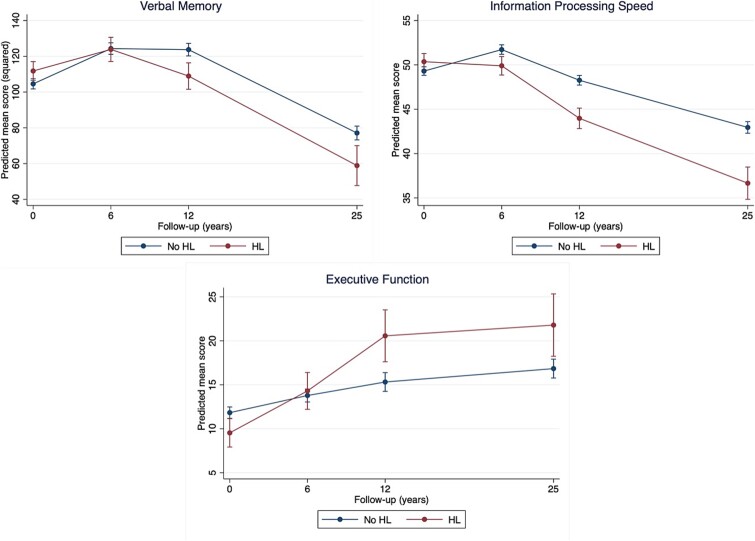
Cognitive trajectories of individuals with baseline hearing loss and those without. Predicted mean scores are estimated marginal means of time by hearing loss (HL) status (HL or no HL) from the fully adjusted linear mixed-effect model with all covariates fixed at their means. For verbal memory and information processing speed, a higher score reflects better performance, whereas for executive function a lower score reflects better performance.

Results for cognitive change over time according to baseline HL status are presented in Table 2 and Figure 1. Participants with HL had significantly better baseline scores on verbal memory and executive function, but significant group-by-time interactions suggested a faster decline over 25 years in all three cognitive domains compared to those without HL. Time-stratified analyses showed that the overall effect was mainly driven by a faster decline from 6 to 12 years in verbal memory (*B* = −14.34, 95% CI −22.15 to −6.54, *P* < .001) and 0 to 12 years in executive function (0 to 6 years: *B* = 2.82, 95% CI 0.32 to 5.32, *P* = .027; 6 to 12 years: *B* = 4.73, 95% CI 1.29 to 8.17, *P* = .007), while information processing speed showed a continuously faster decline.

**Table 2:** Estimated mean difference in baseline cognitive function and change over time in participants with baseline hearing loss and those without

	Baseline		Rate of decline from baseline to 6-year FU		Rate of decline from baseline to 12-year FU		Rate of decline from baseline to 25-year FU		Overall HL by time^a^	
Parameter	Difference	95% CI	Difference	95% CI	Difference	95% CI	Difference	95% CI	*χ* ^2^	*P* value
Verbal memory (*n* = 1811)	7.21^*^	0.89 to 13.53	−7.66^*^	−13.96 to −1.35	−22.00^*^	−29.73 to −14.28	−25.43^*^	−37.07 to −13.79	37.91^*^	<.001
Information processing speed (*n* = 1811)	1.06	−0.04 to 2.16	−2.89^*^	−3.66 to −2.11	−5.35^*^	−6.41 to −4.30	−7.35^*^	−9.25 to −5.45	126.04^*^	<.001
Executive function (*n* = 1798)	−2.29^*^	−4.25 to −0.33	2.82^*^	0.32 to 5.32	7.54^*^	4.24 to 10.85	7.24^*^	3.40 to 11.08	26.02^*^	<.001

Model: HL, time, HL by time, sex, age, age^2^, educational level, LIBRA, LIBRA by time.

*Abbreviations*: HL, hearing loss; CI, confidence interval; LIBRA, LIfestyle for BRAin health index; FU, follow-up.

^a^ *χ* ^2^, 3 degrees of freedom, of interaction between hearing loss (dichotomous) and time (baseline, 6-year, 12-year, 25-year FU).

^*^ *P* value <.05.

#### Hearing loss and incident dementia

Eighty-two (15.8%) individuals with HL and 63 (4.9%) individuals without HL developed incident dementia. The fully adjusted model showed that HL was not significantly associated with higher dementia risk over 25 years (HR = 1.15, 95% CI 0.80 to 1.65, *P* = .445). Cumulative hazard curves by HL status are presented in Supplementary Figure S1.

### Visual loss

#### Visual loss and cognitive decline

**Figure 2:**
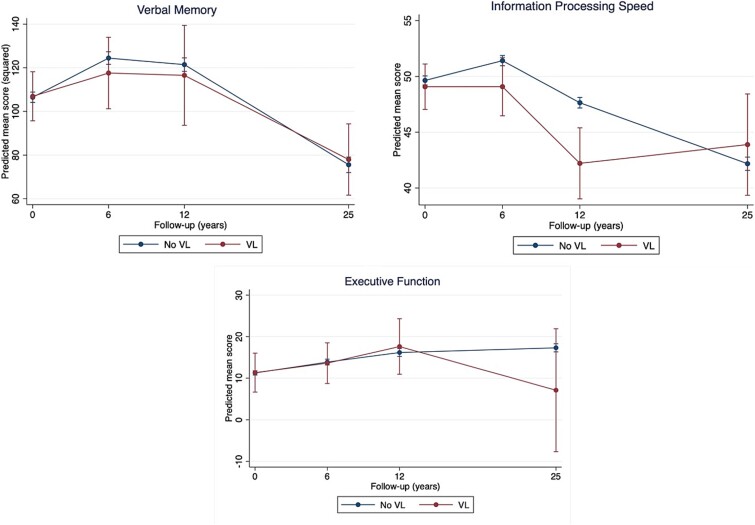
Cognitive trajectories of individuals with baseline visual loss and those without. Predicted mean scores are estimated marginal means of time by visual loss (VL) status (VL or no VL) from the fully adjusted linear mixed-effect model with all covariates fixed at their means. For verbal memory and information processing speed, a higher score reflects better performance, whereas for executive function a lower score reflects better performance.

Table 3 and Figure 2 present the results for cognitive change over time by baseline VL status. At baseline, cognitive test scores did not differ between participants with and without VL. However, group-by-time interactions indicated that participants with VL showed differential cognitive change in information processing speed compared to participants without VL. Specifically, the former showed a significant faster decline from 6 to 12 years (*B* = −3.10, 95% CI −5.94 to −0.26, *P* = .033), followed by improving scores from 12 to 25 years while scores were monotonically declining in those without VL (*B* = 7.14, 95% CI 3.31 to 10.98, *P* < .001). No significant differences were found in verbal memory and executive function. Comparing slopes across time within and across cognitive domains showed inconsistent patterns (Figure 2).

**Table 3:** Estimated mean difference in baseline cognitive function and change over time in participants with baseline visual loss and those without

	Baseline	Rate of decline from baseline to 6-year FU			Rate of decline from baseline to 12-year FU		Rate of decline from baseline to 25-year FU		Overall VL by time^a^	
Parameter	Difference	95% CI	Difference	95% CI	Difference	95% CI	Difference	95% CI	*χ* ^2^	*P* value
Verbal memory (*n* = 1813)	0.44	−10.93 to 11.81	−7.31	−22.40 to 7.77	−5.36	−27.89 to 17.17	1.98	−12.43 to 16.38	2.21	.529
Information processing speed (*n* = 1813)	−0.57	−2.63 to 1.50	−1.77	−3.82 to 0.28	−4.86^*^	−7.88 to −1.85	2.28	−2.19 to 6.75	21.12^*^	<.001
Executive function (*n* = 1800)	0.07	−4.70 to 4.84	−0.33	−6.09 to 5.43	1.37	−5.95 to 8.69	−10.28	−25.78 to 5.21	1.94	.585

Model: VL, time, VL by time, sex, age, age^2^, educational level, LIBRA, LIBRA by time.

*Abbreviations*: VL, visual loss; CI, confidence interval; LIBRA, LIfestyle for BRAin health index; FU, follow-up.

^a^ *χ* ^2^, 3 degrees of freedom, of interaction between VL (dichotomous) and time (baseline, 6-year, 12-year, 25-year).

^*^ *P* value <.05.

#### Visual loss and incident dementia

Survival analysis for VL was not feasible due to the small number of individuals with baseline VL who developed dementia (*n* = 3), resulting in insufficient statistical power.

#### Sensitivity analyses

To test the robustness of the findings for VL, sensitivity analyses used a below-average visual acuity cut-off (<1, *n* = 441) (see Supplementary Table S4, Figure S2). Results for verbal memory were consistent with primary analyses. For information processing speed, the relative improvement in scores from 12 to 25 years that were observed for participants with VL compared to normal vision were replaced by a nonsignificant, i.e. similar, cognitive trajectory in those with and without below-average visual acuity (*B* = −0.37, 95% CI −2.06 to 1.32, *P* = .667). Executive function analyses showed an overall significant interaction effect over time (*χ*^2^ = 19.16, *df* = 3, *P* < .001), with faster decline in those with below-average visual acuity from baseline to 12 years (*B* = 7.97, 95% CI 4.14 to 11.81, *P* < .001) followed by a faster incline from 12 to 25 years (*B* =−4.76, 95% CI −8.88 to −0.63, *P* = .024).

Age group (midlife; *n* = 1328, late life; *n* = 486) did not modify the association between HL and cognitive decline in verbal memory (*P* = .271), information processing speed (*P* = .819) or executive function (*P* = .199). Age-stratified analyses showed no consistent patterns of associations. Similarly, age group did not modify the association between HL and dementia risk (*P* = .053). Examining age group as a modifier in the association between VL and cognitive decline was not feasible due to the small sample sizes.

## Discussion

This prospective cohort study investigated the association between objectively measured HL and VL with cognitive decline and incident dementia over 25 years. Results showed that baseline HL consistently predicted faster decline in all three cognitive domains. Memory and executive functioning declined most from baseline to 12 years, after which the performance gap stabilised, while it continued to widen for information processing speed. Findings were independent of demographics and other modifiable dementia risk factors summarised by LIBRA. Baseline HL was not associated with incident dementia. There was no evidence for a moderating effect of age group (midlife or late life) on the examined associations. Baseline VL initially predicted faster decline in information processing speed but showed inconsistent patterns over time within and across cognitive domains, limiting statistical inferences. Associations with incident dementia could not be examined due to lack of power.

For HL, we found significant associations across all three cognitive domains, which might justify confidence in the observed associations in our sample. Our results are generally supported by earlier studies linking objectively measured HL with cognitive decline [26–29], though few had follow-up periods as long as ours. In a cohort of 1164 older adults, mild (26–40 dB) and moderate to severe (>40 dB) HL were associated with faster cognitive decline over 24 years in global cognition and executive function, but not in verbal memory [29]. Another study involving 15 792 participants found that only moderate to severe HL (>40 dB) was associated with accelerated decline in memory and global cognitive function over 20 years, but not in language and information processing speed [27]. Variations in findings might be due to different cognitive tests used, yet overall, HL appears to be a promising risk factor for cognitive decline. In our study, associations were already present at a 20-dB threshold, which suggests that targeting individuals at risk of HL might be worth studying further. Notably, cognitive decline was most evident and stable in information processing speed, which is believed to be a primary factor and the most sensitive measure of cognitive ageing [30]. Furthermore, results suggest little reason to assume that the observed associations differ between midlife and late life. This is consistent with the Lancet Commission’s report, which indicates that HL is already an important risk factor in midlife [3].

HL was not associated with dementia risk in our study. In contrast, previous meta-analyses consistently report a significant association between the two [6–8]. In the current study, 15.8% of individuals in the HL group developed dementia compared to 4.9% in those without HL, but the higher mean age in the HL group (66.0) compared to the no HL group (45.1) probably explains this. Indeed, after adjusting for demographics and LIBRA, associations were not statistically significant, although they were in the expected direction (HR = 1.15). Reliance on routine care data for dementia diagnosis might have led to non-differential outcome misclassification because of underdiagnosis and some degree of lack of power [31]. Alternatively, HL may relate more to subtle cognitive ageing rather than dementia onset.

No clear association between VL and cognitive decline was found, consistent with some prior research [32, 33]. Others, however, reported a positive association [34–36], supported by systematic reviews and meta-analyses [10–13]. The prevalence of VL in our study was low (3.2%), but similar to the prevalence found in other studies assessing VL using visual acuity with optical correction (3.4% in the UK Biobank Study) [34, 37]. VL in these studies likely represents individuals with VL despite correction (e.g. glasses, lenses), most likely due to uncorrected refractive error, incurable age-related eye conditions like glaucoma, or untreated cataract [5]. Sensitivity analyses showed that below-average visual acuity (<1) was more consistently associated with decline in information processing speed and executive function, suggesting that some form of early or more subtle VL may contribute to cognitive ageing. On the other hand, ~28% of individuals with baseline VL no longer showed VL at follow-ups, possibly due to visual rehabilitation, although we lacked data on this. Future research should investigate how specific eye conditions contribute to cognitive decline and explore the impact of rehabilitative interventions.

Considering public health implications, our findings support the idea of preventing HL for healthy cognitive ageing, in accordance with WHO recommendations [38]. However, whether promoting hearing aids may improve cognition is still a matter of debate. In MAAS, having hearing aids did not affect cognitive decline over 12 years in individuals with HL [39]. Also, the ACHIEVE trial found mixed results, with hearing aids benefitting cognitive performance over 3 years only for people with cardiovascular risk, but not in the overall sample [40]. Regarding VL, while some evidence suggests that visual rehabilitation such as cataract surgery may improve cognitive performances [41], its role in cognitive ageing remains inconclusive and needs further investigation. However, VL is associated with a variety of other adverse health consequences including functional loss [42], reduced quality of life [43] and social isolation [44]. Therefore, preventing and addressing VL should be a public health priority, regardless of its potential impact on cognitive ageing.

Strengths of our study include a large sample, repeated and comprehensive assessments of cognitive functioning over 25 years, objective measures of HL and VL with minimal missing data, and information on relevant confounders. The wide age range allowed us to investigate potential effect modification by age group. The Lancet Commission has identified HL as a midlife risk factor and VL as a late-life risk factor. Therefore, examining associations in cohorts that span both midlife and late life is particularly new and relevant. Main limitations include the possibility of selection bias from dropout of older, less healthy participants. Additionally, the small number of individuals with VL precluded examining its association with dementia incidence. Dementia diagnoses relied mainly on registrations from collaborating general practices, potentially underestimating cases of incident dementia, though efforts were made to contact participants with missing information. Furthermore, cognitive test performances may have been influenced by HL or VL, although these were administered by trained professionals who ensured reliability. Finally, given the non-experimental design, causal inferences cannot be drawn.

In conclusion, we found that HL predicted faster cognitive decline across multiple domains but did not contribute to dementia risk in adults aged 24 to 82 years. No consistent association was found for VL, while below-average visual acuity showed associations with faster decline in information processing speed and executive function. While our findings support that HL is a risk factor contributing to cognitive ageing, its role in dementia risk reduction, as well as the role of VL, needs further research in larger samples.

## Supplementary Material

aa-24-1460-File004_afae271
