# Supplementary material for: Associations of hearing and visual loss with cognitive decline and dementia risk: a 25-year follow-up of the Maastricht Aging Study
Source: Age Ageing. 2024 Dec 18;53(12):afae271. doi: 10.1093/ageing/afae271 (PMC11652612; doi:10.1093/ageing/afae271)
Supplement: aa-24-1460-File004_afae271 [file aa-24-1460-file004_afae271.pdf]

Associations of hearing and visual loss with cognitive decline and dementia risk: A 25-year follow-up of the Maastricht Aging Study (MAAS)

Supplementary Data

Appendix 1. Supplementary methods

1.1 The LIBRA index

Table S1. Operationalization of the LIBRA index in MAAS.

Appendix 2. Supplementary figures and tables

Table S2. Estimated mean difference in baseline cognitive function and change over time in participants with baseline hearing loss and those without: full results

Table S3. Estimated mean difference in baseline cognitive function and change over time in participants with baseline visual loss and those without: full results

Figure S1. Cumulative hazard estimates of incident dementia by baseline hearing loss (HL) status (HL, no HL), adjusted for age (on x-axis), sex, educational level and LIBRA scores

Table S4. Difference in baseline cognitive function and change over time in participants with baseline below-average visual acuity and those without

Figure S2. Cognitive trajectories of individuals with baseline below-average visual acuity and those without

Appendix 1. Supplementary methods

1.1 The LIBRA index

The LIfestyle for BRAin health (LIBRA) index has been well-validated for cognitive functioning/decline [1], incident dementia [2-4], and brain damage [5] in several population-based cohorts. It has also been used as a (surrogate) outcome measure in multidomain prevention trials [6-9]. It is based on the weighted contributions of nine risk factors (physical inactivity, smoking, hypertension, hypercholesterolemia, obesity, diabetes, coronary heart disease, chronic kidney disease, and depression) and three protective factors (high cognitive activity, adherence to the Mediterranean diet, low-to-moderate alcohol consumption) for dementia [10]. The LIBRA score ranges from -5.9 to +12.7, with higher scores indicating an unhealthier lifestyle and greater dementia risk. In MAAS, all LIBRA factors except for Mediterranean diet were available at baseline, resulting in a theoretical range of -4.2 to +12.7. Detailed information on the operationalization of the LIBRA index in MAAS can be found in Table S1.

Table S1. Operationalization of the LIBRA index in MAAS.

**Factor**	**Operationalization**	**Weight**
Coronary heart disease	Presence of cardiac rhythm disorders, chest pain/angina pectoris, heart attack, heart insufficiency, bypass surgery, and/or self-reported presence of heart disease.	+1.0
Diabetes (type-2)	Use of diabetes medication, diabetes diagnosis, and/or self-reported presence of diabetes.	+1.3
Hypercholesterolemia	Use of cholesterol medication, and/or self-reported presence of high cholesterol.	+1.4
Hypertension	Average (of 5 assessments at the research centre) systolic blood pressure ≥ 140 mmHg, and/or average (of 5 assessments at the research centre) diastolic blood pressure ≥ 90 mmHg [11],	+1.6

	and/or current antihypertensive medication use. In case of missings, self-reported presence of hypertension was used.	
Depression	The depression part of the Symptom Check List (SCL 90) was used [12], divided into quartiles. Participants in the highest quartile were assigned in the risk group.	+2.1
Obesity	Body mass index (BMI) ≥ 30 kg/m2 calculated from physical examination at the research centre or (in case of missing BMI) a waist circumference of >88 cm for women and >102 cm for men [13].	+1.6
Smoking	Self-reported current smokers or non-smokers.	+1.5
Low-to-moderate alcohol use	Self-reported alcohol intake. Low to moderate alcohol use was defined as ≤7 alcoholic consumptions per week.	-1.0
Physical inactivity	Self-reported hours per day up and about, divided into tertiles. Participants in the lowest tertile were assigned as physically inactive. In case of missings, self-reported hours per week spent on exercising was used. Participants who did not adhere to the ≥150 minutes per week of exercising were assigned as physically inactive.	+1.1
High cognitive activity	Sum of self-reported average hours per week spent on reading (books, magazines, newspapers) and mind games (chess, checkers, puzzles), divided into tertiles (low/medium/high). Participants in the highest tertile were categorized as cognitively active.	-3.2
Renal dysfunction	Presence of kidney disease (kidney stones excluded).	+1.1
Total theoretical LIBRA range		-4.2 to +12.7

Abbreviations: LIBRA, LIfestyle for BRAin health; MAAS, Maastricht Ageing Study; WHO, World Health Organization.

References

1. Deckers K, Nooyens A, van Boxtel M, et al. Gender and educational differences in the association between lifestyle and cognitive decline over 10 years: The Doetinchem Cohort Study. *Journal of Alzheimer's Disease* 2019;**70**(s1):S31-S41.

2. Deckers K, Barbera M, Köhler S, et al. Long-term dementia risk prediction by the LIBRA score: a 30-year follow-up of the CAIDE study. *International Journal of Geriatric Psychiatry* 2020;**35**(2):195-203.

3. Deckers K, Cadar D, van Boxtel MP, et al. Modifiable risk factors explain socioeconomic inequalities in dementia risk: evidence from a population-based prospective cohort study. *Journal of Alzheimer's disease* 2019;**71**(2):549-557.

4. Vos SJ, Van Boxtel MP, Schiepers OJ, et al. Modifiable risk factors for prevention of dementia in midlife, late life and the oldest-old: validation of the LIBRA index. *Journal of Alzheimer's Disease* 2017;**58**(2):537-547.

5. Heger IS, Deckers K, Schram MT, et al. Associations of the Lifestyle For Brain Health index with structural brain changes and cognition: results from the maastricht study. *Neurology* 2021;**97**(13):e1300-e1312.

6. Deckers K, Köhler S, Ngandu T, et al. Quantifying dementia prevention potential in the FINGER randomized controlled trial using the LIBRA prevention index. *Alzheimer's & Dementia* 2021;**17**(7):1205-1212.

7. van Middelaar T, Hoevenaar-Blom MP, van Gool WA, et al. Modifiable dementia risk score to study heterogeneity in treatment effect of a dementia prevention trial: a post hoc analysis in the preDIVA trial using the LIBRA index. *Alzheimer's research & therapy* 2018;**10**(1):1-10.

8. van Charante EPM, Hoevenaar-Blom MP, Song M, et al. Prevention of dementia using mobile phone applications (PRODEMOS): a multinational, randomised, controlled effectiveness–implementation trial. *The Lancet Healthy Longevity* 2024;**5**(6):e431-e442.

9. Deckers K, Zwan MD, Soons LM, et al. A multidomain lifestyle intervention to maintain optimal cognitive functioning in Dutch older adults—study design and baseline characteristics of the FINGER-NL randomized controlled trial. *Alzheimer's Research & Therapy* 2024;**16**(1):126.

10. Deckers K, van Boxtel MP, Schiepers OJ, et al. Target risk factors for dementia prevention: a systematic review and Delphi consensus study on the evidence from observational studies. *International journal of geriatric psychiatry* 2015;**30**(3):234-246.

11. Chalmers J, MacMahon S, Mancia G, et al. 1999 World Health Organization-International Society of Hypertension Guidelines for the management of hypertension. Guidelines sub-committee of the World Health Organization. *Clinical and experimental hypertension (New York, NY: 1993)* 1999;**21**(5-6):1009-1060.

12. Arrindell W, Ettema J. SCL-90. *Manual for a multidimensional indicator of psychopathology. Lisse: Swets and Zeitlingen* 1986.

13. World Health Organization. Waist circumference and waist-hip ratio: report of a WHO expert consultation. Geneva, 2008.

Appendix 2. Supplementary figures and tables

Table S2. Estimated mean difference in baseline cognitive function and change over time in participants with baseline hearing loss and those without: full results

	**Baseline**		**Rate of decline from baseline to 6-year FU**		**Rate of decline from baseline to 12-year FU**		**Rate of decline from baseline to 25-year FU**		**Overall HL by Time**[a]	
**Parameter**	**Difference**	**95% CI**	**Difference**	**95% CI**	**Difference**	**95% CI**	**Difference**	**95% CI**	χ^2	***P* value**
**Verbal memory (n=1811)**										
Model 1	-42.47*	-48.02 to -36.91	-5.96	-12.37 to 0.45	-19.97*	-27.91 to -12.03	-24.10*	-36.06 to -12.15	30.25*	<0.001
Model 2	7.01*	0.67 to 13.35	-7.91*	-14.29 to -1.52	-21.99*	-29.71 to -14.26	-25.41*	-36.97 to -13.84	38.03*	<0.001
Model 3	7.21*	0.89 to 13.53	-7.66*	-13.96 to -1.35	-22.00*	-29.73 to -14.28	-25.43*	-37.07 to -13.79	37.91*	<0.001
**Information processing speed (n=1811)**										
Model 1	-12.49*	-13.63 to -11.35	-2.85*	-3.63 to -2.06	-5.60*	-6.68 to -4.52	-8.69*	-10.74 to -6.64	129.58*	<0.001
Model 2	0.94	-0.17 to 2.05	-2.90*	-3.68 to -2.12	-5.32*	-6.37 to -4.27	-7.34*	-9.24 to -5.44	126.63*	<0.001
Model 3	1.06	-0.04 to 2.16	-2.89*	-3.66 to -2.11	-5.35*	-6.41 to -4.30	-7.35*	-9.25 to -5.45	126.04*	<0.001
**Executive function (n=1798)**										
Model 1	6.38*	4.91 to 7.84	2.08	-0.43 to 4.59	6.45*	3.12 to 9.79	6.92*	2.72 to 11.12	17.85*	<0.001
Model 2	-2.22*	-4.21 to -0.23	2.78*	0.25 to 5.32	7.47*	4.14 to 10.81	7.16*	3.30 to 11.01	25.09*	<0.001
Model 3	-2.29*	-4.25 to -0.33	2.82*	0.32 to 5.32	7.54*	4.24 to 10.85	7.24*	3.40 to 11.08	26.02*	<0.001

Model 1 (crude) = hearing loss, time, hearing loss by time. Model 2 = Model 1 + sex, age, age^2, educational level. Model 3: Model 2 + LIBRA, LIBRA by time. Abbreviations: HL, hearing loss; CI, confidence interval; LIBRA, LIfestyle for BRAin health index; FU, follow-up. **P* value <0.05. [a] χ^2, 3 degrees of freedom of interaction between hearing loss (dichotomous) and time (baseline, 6-year, 12-year, 25-year).

Table S3. Estimated mean difference in baseline cognitive function and change over time in participants with baseline visual loss and those without: full results

	Baseline		Rate of decline from baseline to 6-year FU		Rate of decline from baseline to 12-year FU		Rate of decline from baseline to 25-year FU		Overall VL by Time[a]	
**Parameter**	**Difference**	**95% CI**	**Difference**	**95% CI**	**Difference**	**95% CI**	**Difference**	**95% CI**	χ^2	***P* value**
**Verbal memory (n=1813)**										
Model 1	-37.21*	-48.41 to -26.02	-7.43	-22.63 to 7.76	-6.53	-29.15 to 16.10	8.95	-2.65 to 20.54	69.51*	<0.001
Model 2	0.58	-10.76 to 11.93	-7.67	-22.74 to 7.41	-5.53	-28.11 to 17.06	2.72	-10.52 to 15.96	3.39	0.335
Model 3	0.44	-10.93 to 11.81	-7.31	-22.40 to 7.77	-5.36	-27.89 to 17.17	1.98	-12.43 to 16.38	2.21	0.529
**Information processing speed (n=1813)**										
Model 1	-12.34*	-14.68 to -10.00	-1.87	-3.95 to 0.20	-5.52*	-8.59 to -2.45	2.15	-0.76 to 5.06	171.57*	<0.001
Model 2	-0.49	-2.59 to 1.60	-1.83	-3.88 to 0.21	-4.95*	-7.92 to 1.97	2.26	-2.23 to 6.76	22.36*	<0.001
Model 3	-0.57	-2.63 to 1.50	-1.77	-3.82 to 0.28	-4.86*	-7.88 to -1.85	2.28	-2.19 to 6.75	21.12*	<0.001
**Executive function (n=1800)**										
Model 1	7.67*	2.92 to 12.42	-1.22	-6.72 to 4.28	1.64	-5.66 to 8.94	-10.79	-34.81 to 13.24	1.09	0.780
Model 2	-0.01	-4.82 to 4.79	-0.23	-6.01 to 5.55	1.39	-5.91 to 8.69	-10.18	-25.65 to 5.29	1.93	0.586
Model 3	0.07	-4.70 to 4.84	-0.33	-6.09 to 5.43	1.37	-5.95 to 8.69	-10.28	-25.78 to 5.21	1.94	0.585

Model 1 (crude) = visual loss, time, visual loss by time. Model 2 = Model 1 + sex, age, age^2, educational level. Model 3: Model 2 + LIBRA, LIBRA by time. Abbreviations: VL, visual loss; CI, confidence interval; LIBRA, LIfestyle for BRAin health index; FU, follow-up. **P* value <0.05. [a] χ^2, 3 degrees of freedom of interaction between visual loss (dichotomous) and time (baseline, 6-year, 12-year, 25-year)

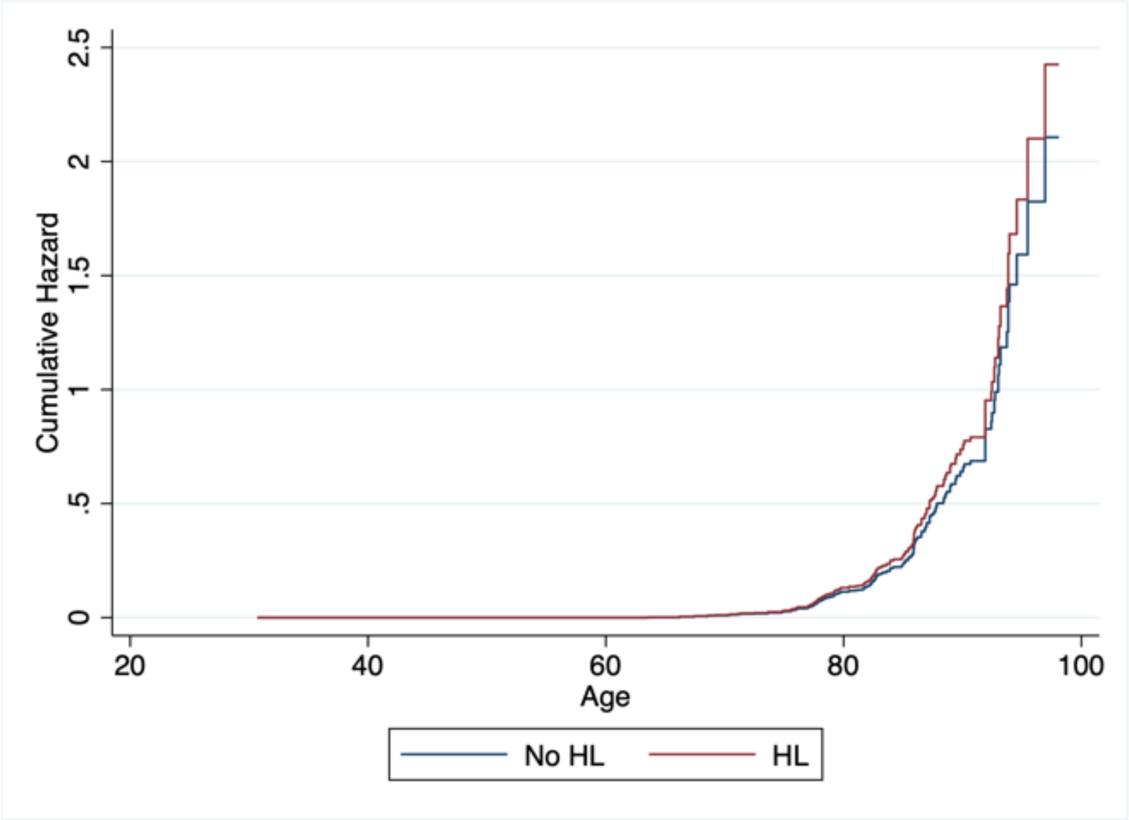

Figure S1. Cumulative hazard estimates of incident dementia by baseline hearing loss (HL) status (HL, no HL), adjusted for age (on x-axis), sex, educational level and LIBRA score.

Table S4. Difference in baseline cognitive function and change over time in participants with baseline below-average visual acuity and those without

	Baseline		Rate of decline from baseline to 6-year FU		Rate of decline from baseline to 12-year FU		Rate of decline from baseline to 25-year FU		Overall Below-average VA by Time[a]	
**Parameter**	**Difference**	**95% CI**	**Difference**	**95% CI**	**Difference**	**95% CI**	**Difference**	**95% CI**	χ^2	***P* value**
**Verbal memory (n=1813)**	-0.70	-6.53 to 5.13	-2.19	-9.13 to 4.75	-4.22	-12.33 to 3.89	-10.02	-22.25 to 2.22	2.91	0.405
**Information processing speed (n=1813)**	-0.66	-1.74 to 0.42	-1.89*	-2.71 to -1.07	-3.90*	-5.00 to -2.80	-4.27*	-6.15 to -2.39	51.71*	<0.001
**Executive function (n=1800)**	-1.16	-2.89 to 0.57	3.26*	0.73 to 5.79	7.97*	4.14 to 11.81	3.22	-0.24 to 6.67	19.16*	<0.001

Model: reduced visual acuity, time, reduced visual acuity by time, sex, age, age^2, education level, LIBRA, LIBRA by time. Abbreviations: VA, visual acuity; CI, confidence interval; LIBRA, LIfestyle for BRAin health index; FU, follow-up; VA, visual acuity. **P* value <0.05. [a] χ^2, 3 degrees of freedom of interaction between reduced visual acuity (dichotomous) and time (baseline, 6-year, 12-year, 25-year).

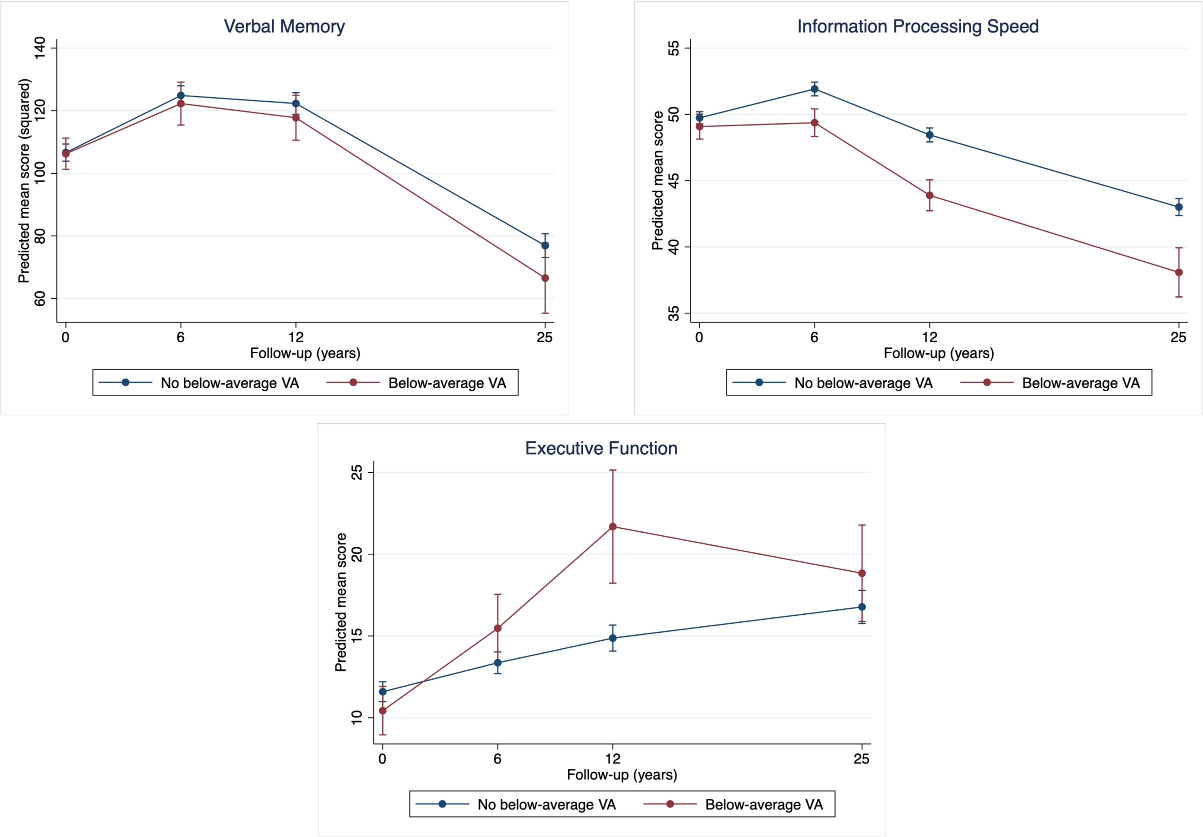

Figure S2. Cognitive trajectories of individuals with baseline below-average visual acuity and those without. Predicted mean scores are estimated marginal means of time by below-average visual acuity (VA) status (below-average VA or no below-average VA) with all covariates fixed at their means. For verbal memory and information processing speed a higher score reflects better performance, whereas for executive function a lower score reflects better performance.
